# Immunometabolic Modulation by Low‐Dose Sodium Butyrate: PI3K/Akt/mTOR Signalling Orchestrates Macrophage Polarization to Protect Against *Clostridioides difficile* Colitis

**DOI:** 10.1111/imm.70179

**Published:** 2026-08-11

**Authors:** Sisi Zhang, Zhou Jingxiang, Caixia Tan, Yuanyuan Xiao, Ju Zou, Biyue Tian, Anhua Wu, Chunhui Li

**Affiliations:** ^1^ Department of Infection Control Center Xiangya Hospital, Central South University Changsha Hunan Province China; ^2^ National Clinical Research Center for Geriatric Disorder Xiangya Hospital Changsha Hunan China; ^3^ Super Drug‐Resistant Organism Infection Prevention and Control Research Center, Xiangya Hospital, Central South University Changsha Hunan Province China

**Keywords:** *Clostridioides difficile* infection (CDI), macrophage, PI3K/AKT/mTOR signalling pathway, sodium butyrate (NaB)

## Abstract

*Clostridioides difficile* infection (CDI) remains a major cause of antibiotic‐associated colitis and is characterized by excessive intestinal inflammation, epithelial barrier disruption and impaired mucosal repair. Although sodium butyrate (NaB), a microbiota‐derived short‐chain fatty acid, has demonstrated anti‐inflammatory and barrier‐protective properties, the immunological mechanisms underlying its protective effects during CDI remain incompletely understood. In this study, we investigated the role of NaB in regulating macrophage polarization and epithelial repair using murine CDI models, primary bone marrow‐derived macrophages, THP‐1‐derived macrophages, Caco‐2 epithelial cells and macrophage–epithelial co‐culture systems. NaB treatment significantly alleviated CDI‐associated weight loss, colonic shortening, histopathological injury and inflammatory cytokine production while restoring epithelial barrier integrity and tight junction protein expression. Mechanistically, NaB promoted macrophage polarization towards an anti‐inflammatory M2 phenotype, characterized by increased CD206 and IL‐10 expression and reduced CD86 and IL‐1β expression. These immunomodulatory effects were accompanied by suppression of PI3K/Akt/mTOR signalling in both colonic tissues and macrophages. Pharmacological inhibition and activation studies further supported the involvement of this pathway in NaB‐mediated macrophage reprogramming. Importantly, conditioned medium from NaB‐treated macrophages enhanced epithelial viability, migration and barrier‐associated protein expression, whereas IL‐10 neutralization partially abolished these protective effects, highlighting a critical role for macrophage–epithelial crosstalk. Collectively, our findings demonstrate that low‐dose NaB protects against CDI‐induced intestinal injury through coordinated regulation of macrophage polarization and epithelial barrier repair, at least in part via modulation of PI3K/Akt/mTOR signalling. These results identify NaB as a promising host‐directed therapeutic strategy for CDI and provide new insights into microbiota‐derived metabolite–immune interactions during intestinal inflammation.

## Graphical abstract

*Clostridioides difficile* toxins TcdA and TcdB enter intestinal epithelial cells (IECs) via specific receptors, disrupt tight‐junction integrity and activate PI3K/Akt/mTOR signalling, promoting inflammasome assembly and IL‐1β release. Toxin‐induced IEC apoptosis further weakens the epithelial barrier. Sodium butyrate (NaB) suppresses PI3K/Akt/mTOR activity and modulates macrophage polarization, shifting M1 pro‐inflammatory responses (IL‐1β, TNF‐α) towards an M2 anti‐inflammatory phenotype (TGF‐β, IL‐10). These coordinated epithelial and macrophage responses illustrate dynamic signalling interactions shaping mucosal inflammation. Activation is indicated by arrows; inhibition is shown by T‐bars.

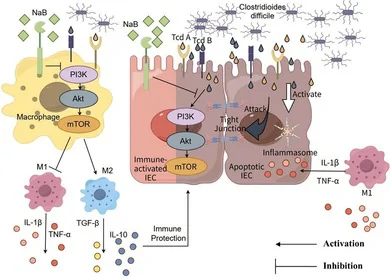

## 1 Introduction

*Clostridioides difficile* ( *C. difficile* ), a Gram‐positive anaerobic bacillus, is the primary cause of antibiotic‐associated diarrhoea and pseudomembranous colitis, especially among hospitalized and older adult patients [1, 2, 3]. Over the past decades, the incidence and recurrence of *C. difficile* infection (CDI) have steadily increased, further complicated by rising antibiotic resistance. Current therapeutic approaches, including antibiotics, faecal microbiota transplantation (FMT) and vaccines, offer partial relief, but recurrence and host immune dysregulation remain key challenges [4, 5, 6, 7]. A deeper understanding of the host‐pathogen interaction is therefore crucial for developing more effective therapeutic strategies.

Despite advances in understanding CDI pathogenesis, critical gaps remain. Most existing therapies focus on bacterial eradication, with limited attention to the restoration of mucosal homeostasis and modulation of host immunity [8]. CDI involves not only direct epithelial injury by bacterial toxins but also excessive immune activation, characterized by cytokine storms and neutrophil infiltration [9]. These factors contribute to disease severity and determine whether colonization remains asymptomatic or evolves into fulminant colitis [10].

Emerging evidence points to the central role of short‐chain fatty acids (SCFAs), especially butyrate, in maintaining intestinal barrier integrity and immune tolerance. Patients with CDI typically exhibit decreased SCFA levels, implicating microbial dysbiosis [11]. Sodium butyrate (NaB), a bioavailable SCFA derivative, has demonstrated efficacy in promoting tight junction expression, limiting inflammation and even directly inhibiting *C. difficile* growth in preclinical models [12]. These findings, coupled with FMT's ability to restore SCFA levels, highlight NaB's potential therapeutic relevance in CDI [13].

In addition, recent studies have revealed that the polarization state of macrophages is integral to intestinal immune responses. While M1 macrophages contribute to inflammation through IL‐1β and IL‐6 secretion, M2 macrophages promote tissue repair by producing IL‐10 and expressing markers such as Arg‐1 and CD206 [14]. NaB has been shown to favour M2 polarization through inhibition of histone deacetylases (HDACs), suggesting that NaB may modulate innate immunity in CDI [15]. Furthermore, the PI3K/Akt/mTOR signalling pathway, known to regulate both inflammation and macrophage function, may serve as a mechanistic bridge between microbial metabolites and immune responses [16]. A comprehensive transcriptome analysis of peripheral blood cells from patients with CDI revealed significant enrichment of gene sets associated with the “PI3K‐Akt signaling pathway” and “mTOR signaling pathway” in gene set enrichment analysis [17]. This finding suggests that the PI3K/Akt/mTOR pathway undergoes dysregulation under the inflammatory conditions observed in vivo. Although its role in CDI remains underexplored, NaB has been reported to downregulate this pathway in other inflammatory diseases.

NaB's immunomodulatory effects are closely linked to its role as a HDAC inhibitor [18]. HDACs regulate chromatin accessibility and transcriptional activation of immune‐associated genes. Recent studies have shown that HDAC inhibition alters the acetylation status of PI3K/Akt/mTOR pathway components, thereby reducing pathway activation and promoting M2‐like macrophage polarization [19]. Conversely, PI3K/Akt/mTOR signalling can modulate HDAC activity, suggesting a bidirectional regulatory loop shaping inflammatory responses [20, 21]. In macrophages, HDAC3 has been shown to regulate mTOR‐dependent metabolic programming, influencing the balance between pro‐inflammatory (M1) and tissue‐repairing (M2) phenotypes [22]. These insights suggest that NaB may reshape macrophage polarization in CDI by modulating HDAC–PI3K/Akt/mTOR crosstalk.

Collectively, these findings suggest that CDI pathogenesis is not solely toxin‐driven but also shaped by SCFA depletion and immune imbalance. NaB thus represents a promising dual‐function agent capable of restoring epithelial integrity and reprogramming macrophage responses via PI3K/Akt/mTOR signalling. In this study, we aim to evaluate the immunoregulatory effects of NaB in a murine CDI model, focusing on macrophage polarization and associated signalling pathways. Our findings are expected to provide new perspectives for host‐directed CDI therapy.

## 2 Materials and Methods

### 2.1 Extraction of Mouse Bone Marrow‐Derived Macrophages (BMDM)

Mice were anaesthetised with sodium pentobarbital (Sinopharm) and euthanized by cervical dislocation. The abdominal skin was disinfected with 75% ethanol, and a midline incision was made to expose the lower abdomen. The colon and proximal small intestine segment of the cecum were excised, placed in ice‐cold PBS and the lumen contents were flushed out using a syringe. Tissues were longitudinally dissected using ophthalmic scissors, rinsed to remove debris and transferred to pre‐digestion buffer (Ca^2+^/Mg^2+^‐free HBSS containing 5% foetal bovine serum, 2 mM EDTA and 0.15 mg/mL DTT). After agitation and centrifugation, samples were washed twice with PBS. Enzymatic digestion was performed in PBS containing 5% foetal bovine serum (Sigma‐Aldrich), 1 mg/mL collagenase VIII (Coolaber) and 0.1 mg/mL DNase I (Coolaber). Supernatants were collected after each digestion cycle. Combined digestion products were filtered through a mesh filter and centrifuged. Cell pellets were resuspended in PBS on ice for subsequent analysis.

### 2.2 Acquisition of *C. difficile* Toxins

BHIS medium (Oxoid) was pre‐warmed under anaerobic conditions, inoculated with a single *C. difficile* colony and adjusted to the appropriate turbidity using the standard turbidity method. The culture reached peak toxin release at 48 h. After adjusting the optical density (OD_600_) value to 0.5, triple replicate cultures were prepared. Following centrifugation, the supernatant was aliquoted, frozen for storage and added at concentrations of 1:10^3^ or 1:10^4^, with a BHIS control group established concurrently.

BHIS (Oxoid) medium was pre‐warmed under anaerobic conditions, inoculated with a single *C. difficile* colony and adjusted to the appropriate turbidity using the turbidity method. The culture reached peak toxin release at 48 h. After adjusting the OD_600_ value to 0.5, triple replicate cultures were prepared. Following centrifugation, the supernatant was aliquoted, frozen for storage and added at concentrations of 1:10^3^ or 1:10^4^, with a BHIS control group established concurrently.

### 2.3 Construction of CDI and NAB‐Fed Mice Models

Five‐week‐old female C57BL/6 mice were selected. All mice received a mixed antibiotic solution in drinking water for 5 consecutive days. This antibiotic mixture contained the following six drugs: ampicillin 200 mg/kg, kanamycin 40 mg/kg, gentamicin 3.5 mg/kg, polymyxin 4.2 mg/kg, metronidazole 21.5 mg/kg and vancomycin 4.5 mg/kg (Aladdin brand). Subsequently, mice were provided sterile water for drinking for 2 days. One day prior to *C. difficile* bacterial solution administration, mice received an intraperitoneal injection of 10 mg/kg clindamycin hydrochloride (Meron). Finally, *C. difficile* R20291 suspension (100 μL, OD600 = 0.3) was administered via gastric tube. Post‐infection, daily monitoring of body weight changes, survival rate, diarrhoea and other relevant symptoms was conducted for approximately 1 week. Additionally, mice in the NaB‐fed group received daily gastric tube administration of 100 mM NaB (Sigma‐Aldrich) throughout the modelling period (from 1 day prior to antibiotic water administration until the end of modelling).

### 2.4 FITC‐Dextran Determination of Intestinal Permeability in Mice

FITC‐dextran (4 kDa; Aladdin) was dissolved at a concentration of 80 mg/mL in dimethyl sulfoxide (DMSO; MP Biomedicals). Mice fasted for 4 h were gavaged with 8 mg FITC‐dextran via gastric tube and euthanized 4 h later. After trimming the whiskers, enucleation was performed on anaesthetised mice. Blood was collected into heparin‐anticoagulated tubes by gently compressing the heart. Serum was separated by centrifugation and stored at −80°C. Thawed serum was centrifuged and diluted 1:50. Concentration was measured by fluorescence detection (excitation wavelength 485 nm; emission wavelength 528 nm), with values interpolated from a standard curve.

### 2.5 Pathologic Scoring of Intestinal Tissues in Mice

Following euthanasia, the intestinal tissues were quickly excised. Representative intestinal segments, including the terminal ileum or colon, were carefully and gently rinsed with pre‐cooled PBS to remove intestinal contents and blood. Thereafter, a small section of undamaged colonic tissues was cut off with ophthalmic scissors and fixed in a neutral buffered formaldehyde fixative (Abiowell). After fixation, tissues were stained with haematoxylin and eosin (HE) and examined under a light microscope. Morphological characteristics of the intestinal tissue, including the morphology of the intestinal villi, integrity of the epithelial cells, structure of the crypts and infiltration of inflammatory cells, were observed under suitable magnification. The acquired microscopic images were scored pathologically according to predetermined scoring criteria (Table 1).

**TABLE 1:** Pathological scoring form for colon injuries.

Scoring indicators	Grading levels and criteria	Scoring instructions
Degree of intestinal villus damage	0: villi intact without atrophy	The structure of the villi is normal and undamaged.
	1: localized slight fusion of villus tips	Slight damage to the tip of the villi, but not affecting the overall structure.
	2: 50% area of villi reduced in height	Damage to the villi is more pronounced, reduced in height, affecting part of the area.
	3: large areas of villi detached to form ulcers	Severe damage to the villi, with large areas of detachment and ulceration.
	4: inflammatory cell infiltration of the whole intestinal wall, loss of crypt structure	Extremely severe damage to the villi, damage to the intestinal wall structure, loss of crypts.
Shedding of epithelial cells	0: no shedding	Epithelial cells are intact with no shedding.
	1 point: small amount of shedding	Small amount of epithelial cell shedding, but does not affect the overall structure.
	2: moderate shedding	Epithelial cells are shedding more, have some effect on the structure of the organization.
	3: large amount of shedding	Large shedding of epithelial cells, severe damage to the tissue structure.
	4: loss of intestinal villi with intestinal necrosis	Extreme shedding of epithelial cells, accompanied by intestinal necrosis.
Degree of structural damage to the crypt fossa	0: normal	Normal crypt structure, no damage.
	1: mild destruction	The structure of the crypt is slightly damaged, but it does not affect the overall function.
	2: moderate destruction	The crypt structure is more obviously damaged, affecting part of the function.
	3: severe destruction	The structure of the crypt is severely disrupted and function is impaired.
	4: loss of crypt structure	Complete loss of crypt structure and function.
Degree of inflammatory cell infiltration	0: no infiltration	No inflammatory cell infiltration.
	1: mild infiltration	A small amount of inflammatory cell infiltration, but not forming aggregates.
	2: moderate infiltration	Inflammatory cell infiltration is more obvious, forming a small number of aggregates.
	3: heavy infiltration	Large infiltration of inflammatory cells, forming obvious aggregates.
	4: inflammatory cell infiltration of the whole intestinal wall	Inflammatory cell infiltration is very severe, involving the entire intestinal wall.

### 2.6 Flow Cytometry

Zombie NIR (Catalogue No.: 423105, Biolegend, dilution: 1:1000) lyophilized dye was reconstituted in DMSO. BMDMs were harvested, washed twice in PBS and pelleted. Cells were stained with Zombie NIR at room temperature in the dark, washed in staining buffer and Fc‐blocked with anti‐CD16/32 (Catalogue No.: 101319, Biolegend, 1 μg/10^6^ cells) on ice. Surface markers, including CD86 (Catalogue No.: 560582, BD Biosciences, 0.5 μg/10^6^ cells), F4/80 (Catalogue No.: 565410, BD Biosciences, 0.5 μg/10^6^ cells), CD11b (Catalogue No.: 557672, BD Biosciences, 0.5 μg/10^6^ cells) and CD45 (Catalogue No.: 103138, BD Biosciences, 0.5 μg/10^6^ cells), were incubated on ice in the dark, washed and fixed in Fixation Buffer (Catalogue No.: 420801, Biolegend). After permeabilization in 1× Intracellular Buffer that was diluted from 10× Intracellular Buffer (Catalogue No.: 421002, Biolegend), cells were stained intracellularly for CD206 (Catalogue No.: 568808, BD Biosciences, 0.5 μg/10^6^ cells), washed twice and resuspended in staining buffer for acquisition.

### 2.7 Western Blotting

Cells (approximately 1 × 10^7^) or intestinal tissues (approximately 1 g) were washed with PBS and lysed on ice using freshly prepared RIPA lysis buffer (Beyotime) containing protease and phosphatase inhibitors. Lysates were centrifuged, and protein concentration was determined using a BCA assay (Biosharp). Protein samples were mixed with 5× SDS loading buffer (Biosharp), denatured and stored at −80°C. Prior to electrophoresis, samples were reheated and centrifuged. After electrophoresis, proteins were transferred onto PVDF membranes. Membranes were blocked in either 5% BSA or 5% non‐fat milk. After blocking, membranes were incubated overnight at 4°C with primary antibodies diluted according to the manufacturer's instructions: PI3K (Catalogue No.: 251221, Zenbio, dilution:1:1000), Akt (Catalogue No.: 60203–2‐lg, Proteintech, dilution:1:5000), mTOR (Catalogue No.: 66888‐1‐lg, Proteintech, dilution:1:5000), p‐PI3K (Catalogue No.: 310164, Zenbio, dilution:1:1000), p‐Akt (Catalogue No.: 310021, Zenbio, dilution:1:1000) and p‐mTOR (Catalogue No.: 381548, Zenbio, dilution:1:1000). Phosphorylated antibodies were diluted in 5% BSA and others in the general antibody diluent. Following primary incubation, membranes were washed with TBST and incubated with HRP‐conjugated secondary antibodies (Catalogue No.: RS0001 and RS0002, Immunoway, dilution:1:10000). After washing, detection was performed using ECL substrate (EpiZyme).

### 2.8 Real‐Time Fluorescence Quantitative PCR

Adherent cells (approximately 5 × 10^6^) or intestinal tissue (approximately 0.5 g) were washed three times with PBS. Total RNA was extracted using Malzol reagent (Magen). After cell lysis and phase separation with chloroform, RNA was precipitated with isopropanol, washed twice with 75% ethanol, air‐dried and dissolved in nuclease‐free water. RNA concentration was measured using a NanoDrop spectrophotometer. After reverse transcription, the reverse transcriptase was inactivated by heating according to the manufacturer's protocol (Table 2). Total RNA was extracted and reverse transcribed into cDNA. Gene‐specific primers were designed based on known sequences and synthesized by a commercial provider. qPCR reactions were performed using 2× SYBR Green Master Mix (SparkJade), primers, diluted cDNA template and nuclease‐free water. The reactions were run on a Step‐One Plus system. Relative mRNA expression was quantified using the 2^−ΔΔCt^ method. ΔCt was calculated as Ct(target) – Ct(reference), and ΔΔCt as ΔCt(treated) – ΔCt(control). Final values represent fold changes in gene expression relative to control samples.

**TABLE 2:** Primer sequence table.

Gene	Forward primer (5′–3′)	Reverse primer (5′–3′)
*Actb*	ACTGCCGCATCCTCCTCCTC	TGCCACAGGATTCCATACCC
*Arg1*	TGCATATCTGCCAAAGACATCG	TCCATCACCTTGCCAATCCC
*Nos2*	CCCTTCAATGGTTGGTACATGG	ACATTGATCTCCGTGACAGCC
*Il10*	CAGAGCCACATGCTCCTAGA	TGTCCAGCTGGTCCTTTGTT
*Il1b*	GCAACTGTTCCTGAACTCAAC	ATCTTTTGGGGTCCGTCAAT
*GAPDH*	GGAGCGAGATCCCTCCAAAAT	GGCTGTTGTCATACTTCTCATGG
*TJP1*	AGCCATTCCCGAAGGAGTTG	ATCACAGTGTGGTAAGCGCA
*OCLN*	GGTCTAGGACGCAGCAGATT	GCCTGGATGACATGGCTGAT

### 2.9 CCK‐8 Assay to Determine Cell Activity

Cells were seeded at 5 × 10^3^ cells/well in 96‐well plates. The outer ring of 36 wells was filled with PBS for humidity maintenance. Blank wells contained medium + CCK‐8 (Abbkine), while control wells contained cells + medium + CCK‐8. Cells were seeded at 5 × 10^3^ cells/well. Cell viability was calculated as follows:

$$\text{Viability}\;\left(\%\right)=\frac{\left(\mathrm{As}-\mathrm{Ab}\right)}{\left(\mathrm{Ac}-\mathrm{Ab}\right)}\times 100$$

where As is the absorbance of experimental wells (cells + medium + CCK‐8 + drug), Ac is the absorbance of control wells (cells + medium + CCK‐8), and Ab is the absorbance of blank wells (medium + CCK‐8).

### 2.10 Cell Scratching Experiment

Ten equidistant lines were pre‐marked on a 6‐well plate for microscopic localization. Caco‐2 cells (1 × 10^6^ cells/well) were inoculated and cultured to 80%–90% confluence. A 10 μL pipette tip was used to create uniform scratches in each well, which were perpendicular to the marked line. After aspirating the culture medium, the wells were washed three times with PBS to remove debris, and then the treatment solution was added. The initial scratched area (0 h) was imaged immediately, and subsequent imaging was performed at 24 and 48 h in the same field of view. The scratch area was quantified using ImageJ, and the healing rate was calculated using the formula:

$$\text{Wound Healing Rate}\;\left(\%\right)=\frac{0h\;\text{area}-24/48h\;\text{area}}{0h\;\text{area}\;}\times 100$$

### 2.11 Caco‐2 Cell and Macrophage Cell Line Conditioned Culture

The THP‐1 monocyte cell line was treated with specific concentrations of drugs in RPMI 1640 medium containing 10% FBS. THP‐1 monocytes were treated with 100 ng/mL PMA for 24 h, followed by replacement with fresh PMA‐free medium and an additional 24 h resting period before experimental stimulation. Differentiation was confirmed morphologically by adherence and spreading, and phenotypically by increased CD11b/CD68 expression. At the same time, Caco‐2 cells were inoculated and DMEM high sugar medium containing 10% FBS was used to establish a monolayer epithelial model under the same culture conditions. A 24‐h culture cycle ensured that the Caco‐2 cells had reached 80%–90% of the growth area, forming a complete tight junction barrier. THP‐1 cell culture medium was subsequently collected and centrifuged, and its supernatant was added to the seeded Caco‐2 cells, which were then cultured for an additional 24 h.

### 2.12 Cell Immunofluorescence

Sterile coverslips were placed in 6‐well plates with complete culture medium. Cells were seeded, allowed to adhere for 24 h, and treated with experimental drugs. The medium was discarded, and fixation was performed with 4% paraformaldehyde (Abiowell), followed by PBS washes. Cells were permeabilized with 0.1% Triton X‐100 (Aladdin) in PBS and washed with 0.05% PBST (Abiowell). Blocking was performed using 1% BSA (Abiowell), and after aspirating the blocking solution, CD206 monoclonal antibody (Catalogue No.: AWA11319, Abiowell, dilution:1:100), CD86 monoclonal antibody (Catalogue No.: AWA02321, Abiowell, dilution:1:100) and Occludin monoclonal antibody (Catalogue No.: AWA10233, Abiowell, dilution:1:100) were added and incubated overnight at 4°C. Secondary antibodies (Goat Anti‐Mouse IgG [HL]—Alexa Fluor 488 [Catalogue No.: RS3208, Immunoway, dilution:1:1000] and Goat Anti‐Rabbit IgG [HL]—Alexa Fluor 647 [Catalogue No.: RS3811, Immunoway, dilution:1:1000]) were applied in 1% BSA and incubated in the dark, followed by 0.05% PBST washes. Nuclei were counterstained with DAPI (Catalogue No.: SL7101, Coolaber, dilution:1:100) in the dark and washed with 0.05% PBST. Coverslips were mounted on slides with anti‐fluorescence quenching encapsulant (Abiowell), sealed and imaged using a Zeiss LSM900 confocal microscope.

### 2.13 IL‐10 Neutralization and IL‐10R Blockade Experiments

Conditioned medium (CM) was collected from THP‐1‐derived macrophages treated with 0.1 mM NaB for 24 h. Caco‐2 cells were incubated with NaB‐treated CM in the presence or absence of an IL‐10‐neutralizing antibody (Catalogue No.: MAB217‐SP, R&D systems), recombinant human IL‐10 (rhIL‐10, Catalogue No.: CX04, Novoprotein), or corresponding isotype controls (Catalogue No.: MV080409, Abinscience). To block IL‐10 signalling, Caco‐2 cells were pretreated with a human IL‐10Rα‐blocking antibody (Catalogue No.: MAB274‐SP, R&D systems) before CM exposure. After 24 h, epithelial wound repair and barrier integrity were evaluated using wound‐healing and barrier‐related proteins.

### 2.14 Statistical Methodology

Statistical analyses were performed using GraphPad Prism 9.5, SPSS 27.0, and Excel. Data are presented as mean ± SD. Group comparisons were conducted using Student's *t*‐tests or one‐way ANOVA for normally distributed data, with nonparametric tests applied when normality assumptions were not met.

## 3 Results

### 3.1 NaB Ameliorates Intestinal Inflammatory Injury in CDI Mice

Following CDI, mice exhibited significant weight loss (*p* < 0.01, *p* < 0.05) and diarrhoea, all of which were attenuated by NaB treatment (Figure 1A–F). Histological analysis confirmed acute colitis with epithelial erosion, crypt distortion and inflammatory infiltration exclusively in CDI mice (*p* < 0.05); NaB preserved mucosal architecture and significantly reduced injury scores (*p* < 0.05), while CDI‐induced colon shortening was reversed by NaB (6.12 ± 0.58 cm vs. 7.30 ± 0.60 cm, *p* < 0.05), which alone modestly increased colon length (8.46 ± 0.64 cm vs. 7.40 ± 0.30 cm, *p* < 0.01) (Figure 1G–J). Mice infected with CDI exhibited significantly increased intestinal permeability (*p* < 0.0001). Intervention with NaB (*p* < 0.0001) effectively alleviated intestinal mucosal damage and reduced intestinal permeability (Figure 1K). Expression of tight junction proteins ZO‐1 (*p* < 0.001, *p* < 0.0001) and Occludin (*p* < 0.0001, *p* < 0.0001) was significantly reduced in CDI mice and restored by NaB; NaB alone (*p* < 0.0001) enhanced their expression beyond baseline (Figure 1L–N). These results demonstrate that NaB maintains epithelial barrier integrity, mitigating mucosal damage and promoting colonic homeostasis in CDI.

**FIGURE 1:**
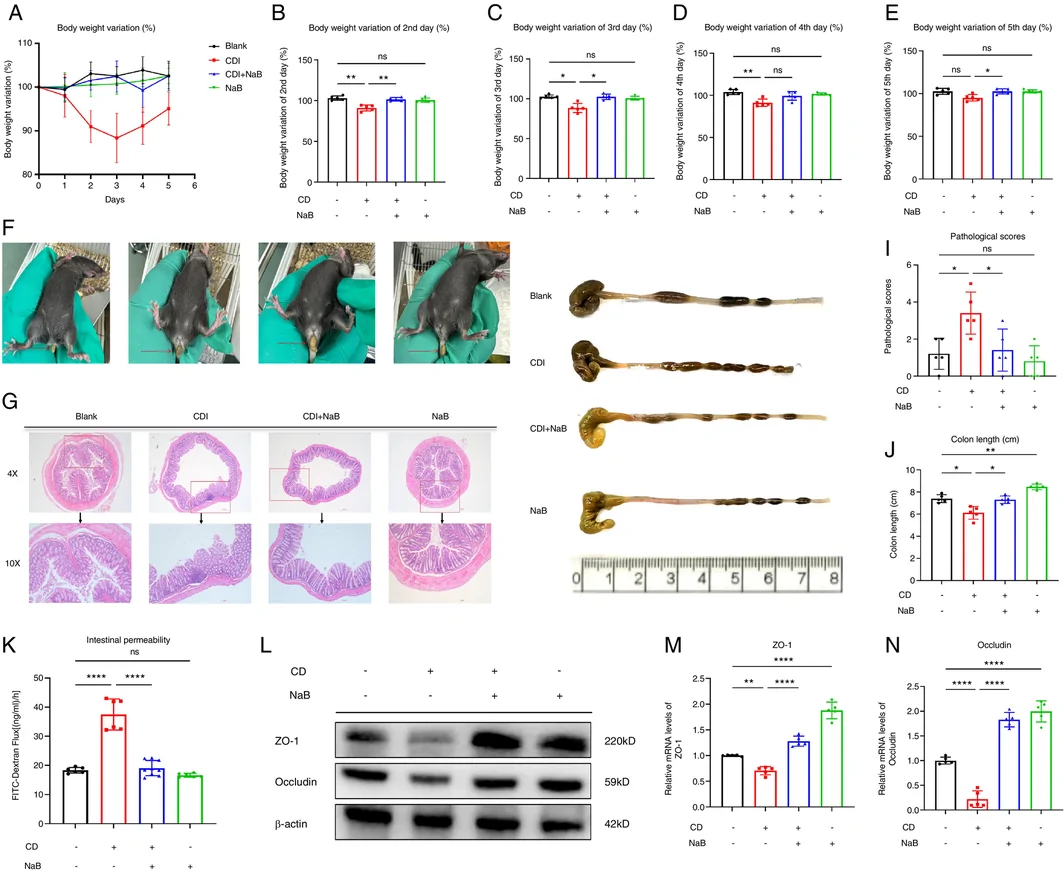
NaB ameliorates intestinal inflammatory injury in CDI mice. NaB treatment markedly attenuated body weight loss, diarrhoea and colonic shortening in CDI mice, indicating protective effects on mucosal integrity. (A–E) Daily and cumulative body weight changes in Blank, CDI, CDI + NaB and NaB groups; (F) Representative faecal morphology; (G) Haematoxylin and eosin (H&E) staining of colonic sections at low (4×) and high (10×) magnifications; (H‐J) Quantification of colonic length and histopathological scores; (K) Intestinal permeability determined by FITC‐dextran assay; (L–N) Western blot and qRT‐PCR analysis of tight junction proteins ZO‐1 and Occludin in colonic tissues. Data are presented as mean ± SD (*n* = 5).

### 3.2 Effect of NaB on the Activity of *C. difficile* Supernatant‐Intervened Caco‐2 Cells

Low‐dose NaB (0.1–1 mM) exhibited negligible toxicity in Caco‐2 cells (*p* = ns) (Figure 2A,B). The cell viability of Caco‐2, THP‐1‐derived macrophages, RAW264.7 and BMDM and the tight junction protein expression of Caco‐2 upon graded NaB exposure are shown in (Figure S1). *C. difficile* culture supernatant (1:1000) induced apoptosis and impaired viability over 24–72 h; these effects were significantly mitigated by 0.1 and 0.5 mM NaB (*p* < 0.01, *p* < 0.01), respectively (Figure 2C–E). Wound‐healing assays demonstrated that NaB (0.1 and 0.5 mM) enhanced Caco‐2 migration and restored closure rates impaired by *C. difficile* supernatant (Figure 2F–I). Correspondingly, *C. difficile* supernatant reduced ZO‐1 (*p* < 0.001, *p* < 0.05) and Occludin (*p* < 0.05, *p* < 0.0001) expression at protein and mRNA levels, which were upregulated by NaB co‐treatment; NaB alone (*p* < 0.001) also increased tight junction protein expression relative to controls (Figure 2J–L). These data indicate that low‐dose NaB selectively preserves epithelial barrier function by promoting cell viability, migration and tight junction integrity under *C. difficile* ‐induced stress.

**FIGURE 2:**
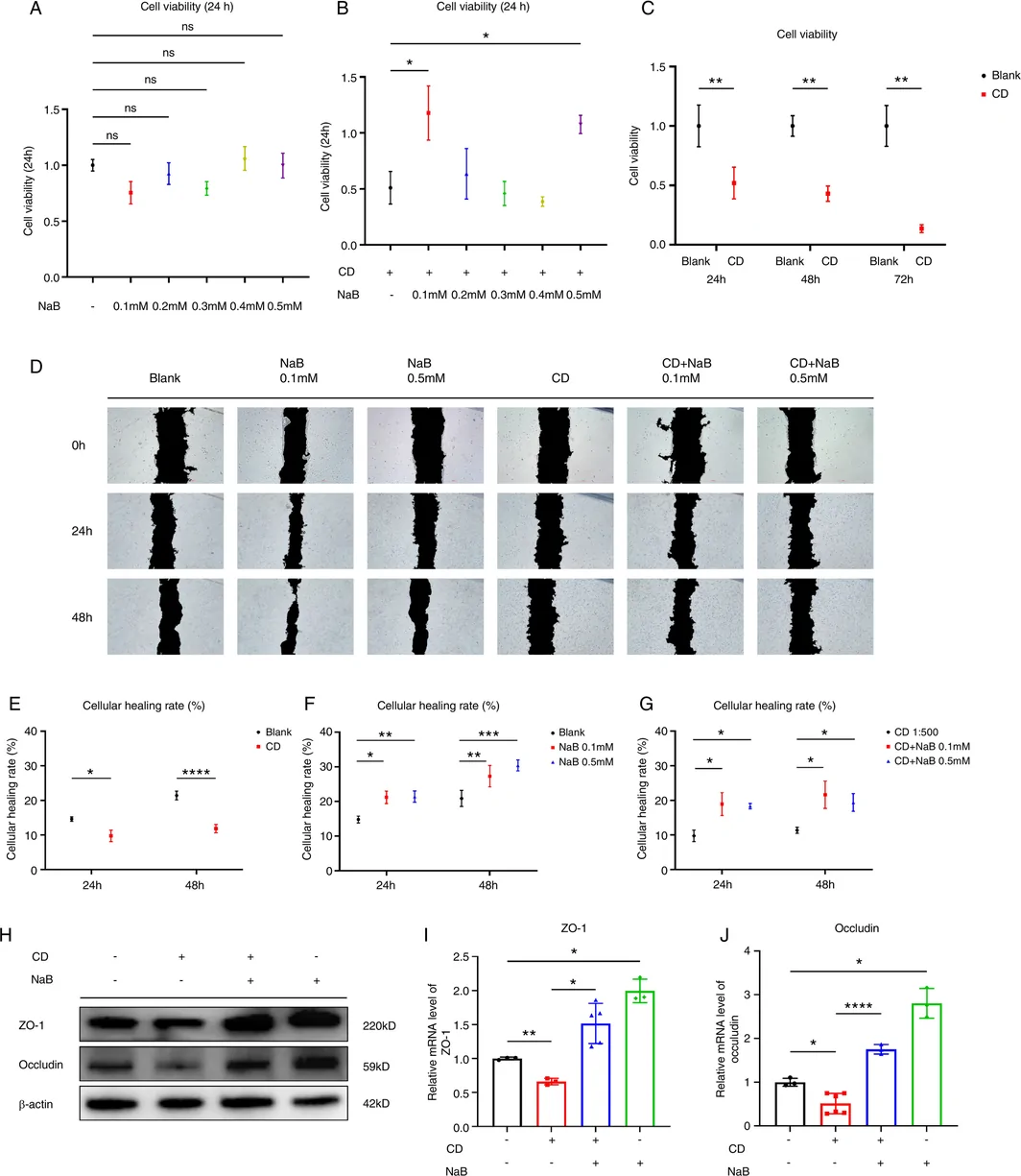
Effect of NaB on the activity of *C. difficile* supernatant‐intervened Caco‐2 cells. NaB treatment enhanced epithelial cell viability and migration under *C. difficile–induced* stress. (A) Viability of Caco‐2 cells after 24 h exposure to NaB (0.1–1 mM); (B–C) Viability of cells treated with *C. difficile* supernatant (1:1000) with or without NaB co‐treatment after 24, 48, and 72 h; (D–G) Representative wound‐healing images and quantitative migration rates at 0, 24, and 48 h; (H–J) Western blot and qRT‐PCR analysis of ZO‐1 and Occludin expression. Data are shown as mean ± SD (*n* ≥ 3).

### 3.3 NaB Modulates Macrophage Polarization Trends in CDI

Flow cytometric analysis of colonic lamina propria macrophages (CD11b^+^F4/80^+^) revealed that CDI decreased the M2/M1 ratio (CD206^+^/CD86^+^), which was restored by NaB supplementation; NaB alone elevated this ratio above baseline (Figure 3A). Similarly, BMDM exposed to *C. difficile* supernatant showed reduced M2/M1 polarization that was reversed by NaB co‐treatment (Figure 3B). Confocal imaging confirmed that NaB restored CD206 expression significantly suppressed (*p* < 0.001) by *C. difficile* exposure (Figure 3C,D). qPCR analysis in BMDMs and RAW 264.7 cells demonstrated that *C. difficile* decreased IL‐10 and increased IL‐1β and iNOS expression, whereas NaB rescued IL‐10 while suppressing pro‐inflammatory markers (Figure 3E–N). Collectively, these findings indicate that NaB promotes an anti‐inflammatory M2 macrophage phenotype by enhancing CD206 and IL‐10 while inhibiting M1‐associated cytokines.

**FIGURE 3:**
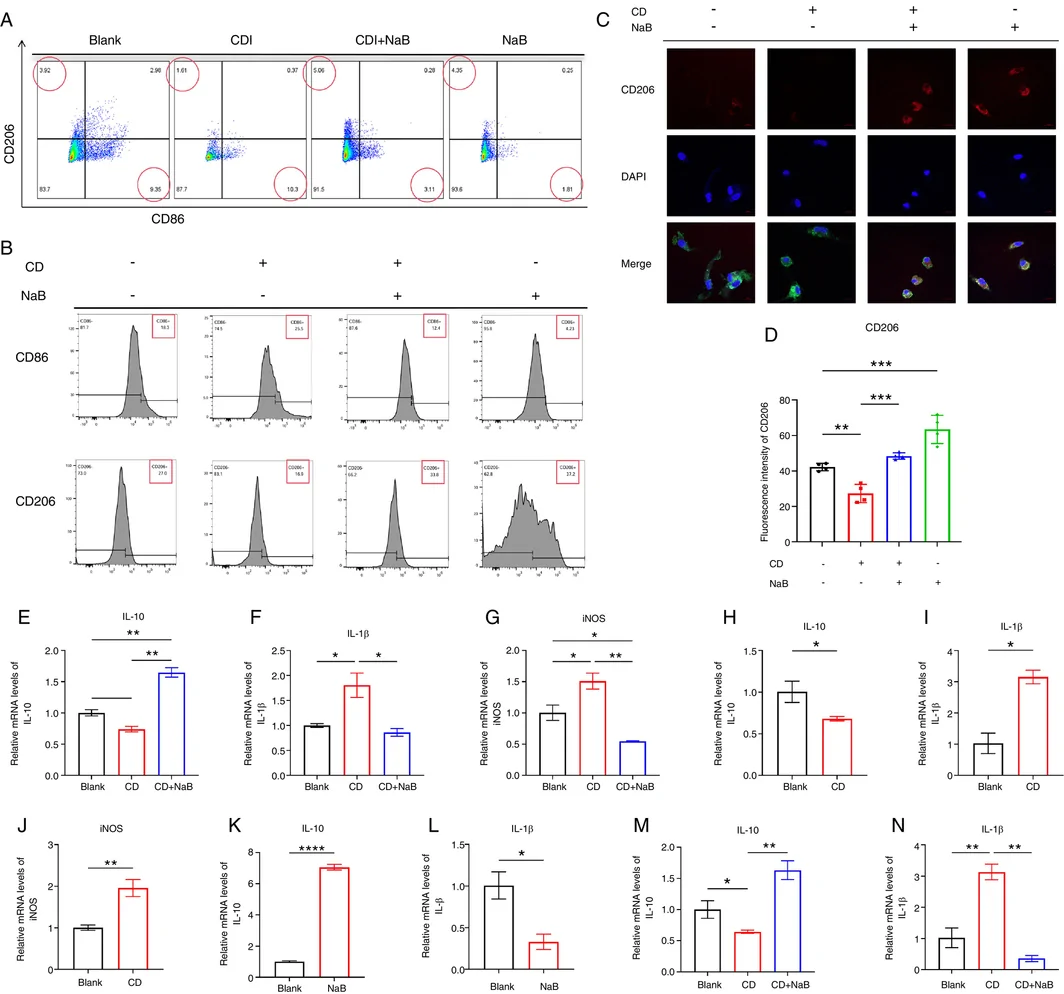
NaB modulates macrophage polarization trends in CDI. NaB enhanced the M2/M1 macrophage ratio and suppressed pro‐inflammatory cytokine expression following CDI. (A, B) Flow cytometric plots of colonic macrophages and bone marrow‐derived macrophages (BMDMs); (C, D) Immunofluorescence staining and quantification of CD206 (M2 marker) in BMDMs (scale bar = 10 μm); (E–N) qRT‐PCR analysis of IL‐10, IL‐1β and iNOS mRNA expression in BMDMs and RAW264.7 cells. CD206 (red), CD11b (green), DAPI (blue) and merge (Merge) channels and merge images show co‐localization of fluorescent signals, and DAPI staining indicates a uniform and intact distribution of nuclei.

### 3.4 NaB Inhibits Activation of the PI3K/Akt/mTOR Pathway

CDI increased colonic p‐PI3K, p‐Akt and p‐mTOR levels in vivo, indicating pathway activation, which was suppressed by oral NaB (Figure 4A). In vitro, NaB (0.1–1 mM) reduced PI3K/Akt/mTOR phosphorylation in Caco‐2 and RAW264.7 cells, with maximal effects at 0.1 and 0.5 mM (Figure 4B,C). Pharmacologic modulation with LY294002 (PI3K pathway inhibitor) or 740Y‐P (PI3K pathway agonist) confirmed that 0.1 mM (*p* < 0.001) and 0.5 mM (*p* < 0.05) NaB attenuate *C. difficile* supernatant–induced cytotoxicity by limiting pathway hyperactivation, respectively (Figure 4D–F). NaB and LY294002 also rescued impaired migration and restored Occludin expression under *C. difficile* or 740Y‐P challenge (Figure 4G–J). Together, these findings demonstrate that NaB preserves epithelial integrity during CDI by inhibiting PI3K/Akt/mTOR signalling and promoting barrier repair.

**FIGURE 4:**
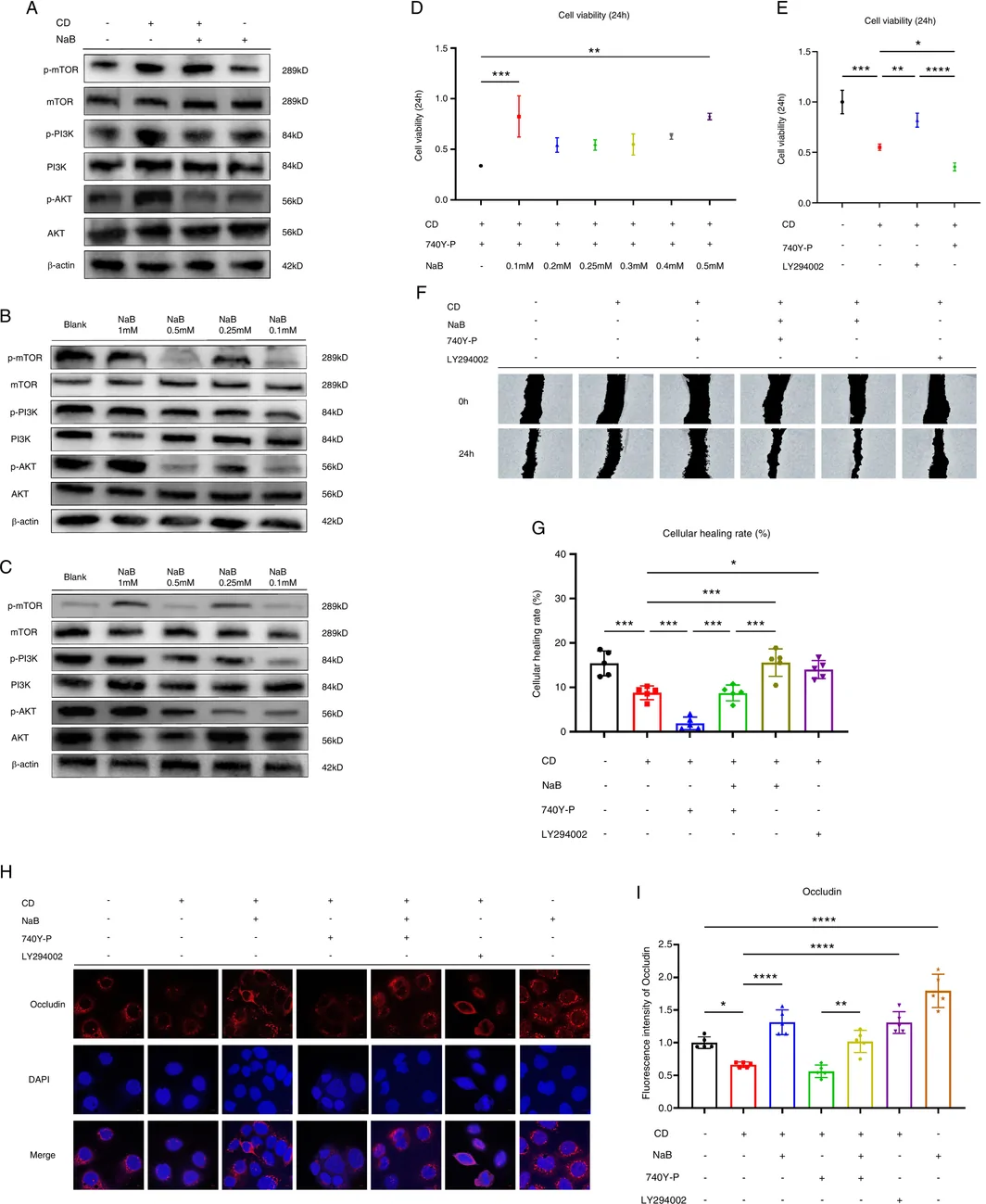
NaB inhibits activation of the PI3K/Akt/mTOR pathway. NaB downregulated phosphorylation of PI3K, Akt and mTOR, indicating inhibition of the PI3K/Akt/mTOR pathway. (A) Western blot of total and phosphorylated PI3K, Akt and mTOR in colonic tissues of Blank, CDI, CDI + NaB and NaB groups; (B, C) Protein expression in Caco‐2 and RAW264.7 cells treated with NaB (0.1–1 mM); (D–G) Viability of Caco‐2 cells and wound scratch healing upon combined treatment with the PI3K/Akt pathway agonist 740Y‐P (10 μM) and inhibitor LY294002 (10 μM); (H, I) Immunofluorescence imaging and quantification of Occludin (scale bar = 10 μm). Occludin (red), DAPI (blue) and merge (Merge) channels. Occludin (red), DAPI (blue) and merge (Merge) channels. Merge images show co‐localization of fluorescent signals, and DAPI staining indicates a uniform and intact distribution of nuclei. In addition, LY294002 was continuously present throughout the entire treatment period, including during macrophage stimulation and subsequent co‐culture experiments.

### 3.5 NaB Acts on the PI3K/Akt/mTOR Pathway to Regulate M2/M1

Confocal microscopy of BMDMs revealed that *C. difficile* supernatant reduced CD206 and increased CD86 expression, indicative of M1 polarization; these effects were reversed by NaB or LY294002, while 740Y‐P further enhanced M1 markers. Notably, NaB co‐treatment with 740Y‐P restored CD206 and suppressed CD86 expression (Figure 5A–C), even under 740Y‐P stimulation (Figure 5D). In RAW264.7 cells, 740Y‐P downregulated Arg and upregulated CD86, both reversed by NaB (Figure 5E). qPCR showed opposing effects of 740Y‐P and LY294002 on IL‐10 and IL‐1β, respectively, which were normalized. Flow cytometry confirmed that NaB and LY294002 elevated the M2/M1 ratio by NaB (Figure 5F–I). These findings identify PI3K/Akt/mTOR as a key regulatory axis through which NaB promotes anti‐inflammatory M2 polarization.

**FIGURE 5:**
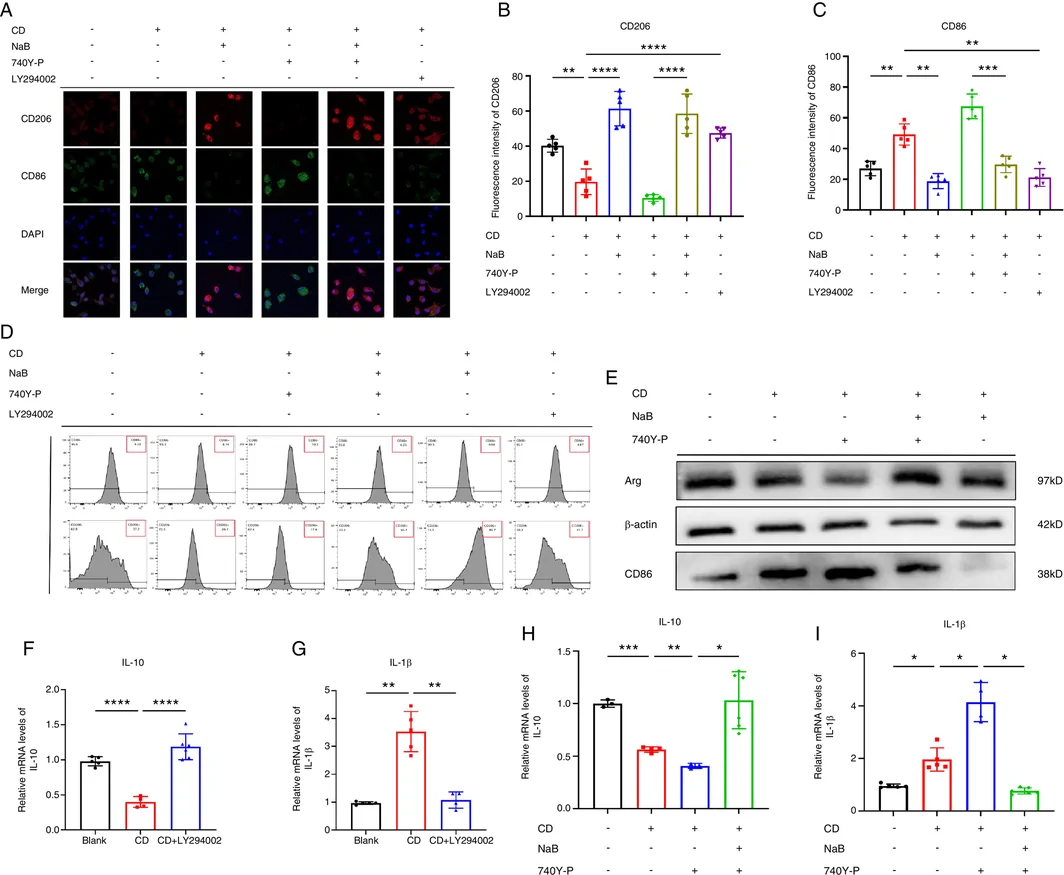
NaB acts on the PI3K/Akt/mTOR pathway to regulate M2/M1. NaB promoted M2 polarization and inhibited M1 activation via PI3K/Akt/mTOR inhibition. (A–C) Immunofluorescence images and quantification of CD206 (M2) and CD86 (M1) in BMDMs treated with NaB (0.1 mM), LY294002 (10 μM) and 740Y‐P (10 μM) (scale bar = 10 μm); (D) Flow cytometric plots of macrophage subsets; (E) Western blot analysis of Arg1 (M2) and CD86 (M1) expression in RAW264.7 cells; (F–I) qRT‐PCR analysis of IL‐10 and IL‐1β mRNA in macrophages under the same treatments. CD206 (red), CD86 (green), DAPI (blue) and merge (Merge) channels. Merge images show co‐localization of fluorescent signals, and DAPI staining indicates a uniform and intact distribution of cell nuclei (Figure 6A–C).

### 3.6 Elevated M2/M1 With NaB Intervention is Protective of the Intestinal Barrier

Caco‐2 and THP‐1 co‐culture models were used to assess whether NaB promotes epithelial barrier protection via macrophage polarization. Prior to subsequent experiments, the complete differentiation of THP‐1 monocytes into macrophages was verified (Figure S2).

In monoculture, *C. difficile* supernatant reduced Caco‐2 viability, which was rescued by 0.1 or 0.5 mM NaB and by PI3K/Akt/mTOR inhibition. Further, 740Y‐P impaired viability, an effect attenuated by NaB (Figure 6A–C). Scratch assays showed that NaB enhanced wound closure under both basal and *C. difficile*‐challenged conditions, with greater effects observed in conditioned co‐cultures. Notably, NaB‐treated conditioned cultures exhibited accelerated healing compared to monoculture, suggesting that macrophage‐derived factors, likely IL‐10, potentiate NaB's reparative effects (Figure 6D–H). qPCR analysis confirmed that NaB upregulated ZO‐1 and Occludin transcripts in conditioned Caco‐2 cells (Figure 6I–J). These findings indicate that NaB enhances epithelial viability, migration and tight junction expression, effects amplified by macrophage‐derived anti‐inflammatory mediators via M2/M1 polarization.

**FIGURE 6:**
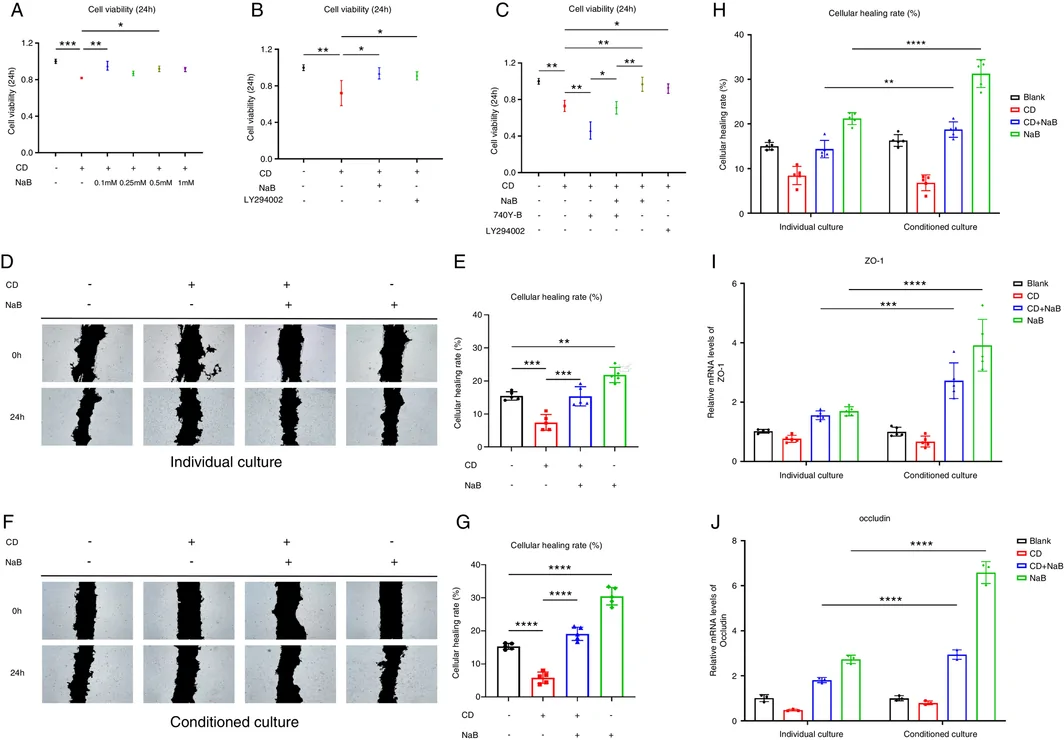
Elevated M2/M1 with NaB intervention is protective of the intestinal barrier. NaB restored epithelial barrier integrity and cell viability under *C. difficile‐induced* injury in both monoculture and macrophage co‐culture systems. (A–C) Cell viability of Caco‐2 cells treated with NaB (0.1–1 mM), LY294002 (10 μM) and 740Y‐P (10 μM), alone or combined; (D–H) wound‐healing assays and quantification in Caco‐2 monocultures and THP‐1 co‐cultures; (I–J) qRT‐PCR analysis of ZO‐1 and Occludin mRNA after 24 h.

To determine whether macrophage‐derived IL‐10 contributes to epithelial protection, Caco‐2 cells were subsequently exposed to conditioned medium (CM) derived from NaB‐treated macrophages in the presence or absence of IL‐10 neutralization or IL‐10 receptor blockade. Epithelial wound repair and barrier integrity were evaluated using wound‐healing assays and analysis of barrier‐related proteins. Compared with the CD group, NaB‐treated CM significantly accelerated wound closure and restored Occludin expression. These beneficial effects were markedly attenuated by IL‐10 neutralization and were similarly diminished following IL‐10Rα blockade. In contrast, supplementation with recombinant human IL‐10 (rhIL‐10) partially rescued the impaired epithelial repair and barrier restoration caused by IL‐10 neutralization. Notably, isotype control antibodies had no appreciable effect on CM‐mediated epithelial protection (Figure S3). Together, these findings indicate that macrophage‐derived IL‐10 is a critical mediator of the pro‐repair and barrier‐protective effects induced by NaB‐treated macrophages.

## 4 Discussion

Our findings demonstrate that NaB ameliorates CDI‐induced colonic injury through coordinated effects on epithelial barrier preservation and immune modulation. In the present study, NaB treatment was associated with improved epithelial integrity, enhanced tight junction protein expression, attenuation of inflammatory responses and promotion of an anti‐inflammatory macrophage phenotype. Mechanistically, these protective effects were accompanied by modulation of PI3K/Akt/mTOR signalling, suggesting that this pathway may participate, at least in part, in the beneficial actions of NaB during CDI. Furthermore, the protective effects of NaB were attenuated following macrophage depletion or pharmacological reactivation of PI3K/Akt/mTOR signalling, highlighting the potential importance of macrophage–epithelial crosstalk in mediating intestinal protection.

In our CDI mouse model, NaB treatment mitigated weight loss, colon shortening and histopathological damage. Additionally, it reduced pro‐inflammatory IL‐1β while increasing anti‐inflammatory IL‐10, highlighting its anti‐inflammatory capacity. These findings align with reports linking reduced faecal SCFAs to CDI severity and NaB's ability to attenuate CDI‐induced colitis by modulating bile acid metabolism and enhancing tight junction proteins via HIF‐1α [23, 24]. NaB's anti‐inflammatory mechanisms likely involve suppression of NF‐κB and MAPK pathways, inhibition of immune cell overactivation and antioxidant effects, collectively alleviating intestinal inflammation [25, 26].

In vitro, *C. difficile* supernatant impaired Caco‐2 cell viability, migration and tight junction protein expression (e.g., Occludin, ZO‐1). Notably, NaB treatment effectively reversed these detrimental effects within the concentration range tested (0.1–1 mM), thereby improving epithelial cell survival and barrier integrity. The protective effects of butyrate on enter intestinal epithelial cells (IECs) have been widely documented, particularly through enhancement of tight junction protein expression and maintenance of epithelial barrier function. Previous studies have demonstrated that butyrate upregulates Claudin‐1 and ZO‐1 expression while supporting epithelial survival signalling pathways, including Akt activation [27].

Importantly, we further performed supplementary in vitro dose–response analyses under identical experimental conditions. These experiments demonstrated that NaB concentrations within the relatively low range of 0.01–1 mM exerted the most stable protective effects on epithelial viability, inflammatory regulation and barrier‐associated protein expression. In contrast, higher concentrations progressively reduced cellular viability and suppressed normal cellular activity. Specifically, NaB concentrations above 5 mM showed evidence of impaired cellular tolerance, whereas the statistically significant increase in cell viability at 0.1 mM NaB in THP‐1‐derived macrophages. These findings support the rationale underlying our experimental concentration selection. Protective effects were observed predominantly within the low‐concentration range tested and further indicate that the biological effects of NaB are highly concentration‐dependent under the present experimental conditions. This concentration selection was also intended to minimize the potential pro‐apoptotic and excessive HDAC inhibitory effects previously reported at higher butyrate concentrations. Indeed, high‐dose butyrate exposure has been associated with cell‐cycle arrest, impaired epithelial proliferation and altered cellular metabolic activity, while several studies have further suggested that excessive butyrate concentrations may influence *C. difficile* physiological behaviour, including sporulation dynamics [28, 29]. Collectively, these observations imply that low‐concentration NaB may exert protective effects through mechanisms that are at least partially distinct from classical high‐dose HDAC inhibition, although the precise molecular basis underlying this concentration‐dependent functional divergence warrants further investigation.

In addition, although the in vivo NaB gavage concentration (100 mM) appears substantially higher than the concentrations used in vitro, these values are not directly comparable because orally administered NaB undergoes rapid dilution, microbial metabolism, intestinal absorption and colonocyte utilization before reaching local tissue targets [30]. Therefore, relatively high luminal concentrations are commonly employed in experimental intestinal inflammation models to achieve biologically effective exposure within the intestinal microenvironment. Of note, the 100 mM concentration used in the present study refers to the concentration of the gavage solution rather than the final tissue concentration achieved in vivo. Nevertheless, we acknowledge that comprehensive in vivo dose‐escalation and pharmacodynamic studies remain necessary to further define the optimal therapeutic window and safety profile of NaB in CDI‐associated intestinal inflammation.

Macrophage polarization is pivotal in regulating inflammation and tissue repair in intestinal diseases like CDI [31]. M2 macrophages, marked by Arg1 and CD206, produce IL‐10 and TGF‐β to aid healing, while M1 macrophages drive inflammation via IL‐1β and TNF‐α [32]. In our CDI mouse model and in vitro studies, NaB shifted polarization towards M2, increasing CD206 and reducing CD86 expression. NaB's pro‐M2 effects are linked to STAT6 signalling, HDAC3 inhibition and possibly mTOR‐mediated autophagy and metabolic reprogramming [33, 34]. Our results show that NaB elevates the M2/M1 ratio in CDI, decreasing pro‐inflammatory cytokines (IL‐1β, TNF‐α) and boosting tissue repair factors (TGF‐β, IL‐10) [35]. Notably, primary BMDMs exhibited greater sensitivity to *C. difficile* supernatant toxicity than cell lines (e.g., RAW264.7, Caco‐2), suggesting cell‐specific responses that merit further study.

A central finding is NaB's inhibition of the PI3K/Akt/mTOR pathway. In colonic tissue and macrophages, NaB reduced phosphorylation of PI3K, Akt and mTOR. This pathway governs macrophage polarization—mTORC1 inhibition favours M2, while Akt isoforms exert opposing effects (Akt1 pro‐M2, Akt2 pro‐M1) [36]. We propose NaB selectively modulates Akt isoforms to promote M2 polarization, though this requires confirmation. Using PI3K inhibitor LY294002 and activator 740Y‐P, we validated that pathway inhibition enhances NaB's pro‐M2 effects, while activation diminishes them. This positions PI3K/Akt/mTOR as a key mediator of NaB's immunomodulatory role in CDI, distinct from STAT6‐dependent mechanisms. Additionally, in another GEO accession GSE155167 (whole transcriptome data of *C. difficile* strain 630), expression trends of genes in the PI3K/Akt/mTOR pathway (PIK3CA, AKT1, MTOR, etc.) were observed in host IEC models between the infected and treated groups. This provides supplementary evidence for the in vitro experiments and is highly consistent with the in vitro results of this study [37].

However, the mechanistic interpretation of these findings should be approached with appropriate caution. Although LY294002 is widely used as a classical PI3K inhibitor, it is also recognized as a first‐generation reversible inhibitor with documented off‐target effects on several kinases, including mTOR, DNA‐PK and CK2. Therefore, pharmacological inhibition alone cannot definitively establish pathway‐specific causality [38, 39]. Moreover, because LY294002‐mediated inhibition is reversible, sustained pathway suppression may depend on continuous exposure conditions [40]. We fully acknowledge that the absence of genetic approaches, such as siRNA‐mediated knockdown or gene‐specific perturbation, limits the mechanistic specificity of the present study. Accordingly, our findings should be interpreted as demonstrating that NaB‐mediated protective effects are associated with modulation of PI3K/Akt/mTOR signalling rather than conclusively proving direct pathway dependence. Future studies employing RNA interference, gene‐editing strategies, or isoform‐specific targeting approaches will be necessary to more rigorously establish the causal contribution of PI3K/Akt/mTOR signalling to NaB‐mediated macrophage polarization and epithelial protection.

M2 macrophages bolster intestinal barrier integrity by secreting IL‐10 and TGF‐β and upregulating tight junction proteins [41]. In our CDI model, NaB‐driven M2/M1 ratio increases correlated with reduced disease severity and faster recovery. Co‐culture of Caco‐2 and THP‐1 cells confirmed that NaB‐induced M2 polarization enhanced IEC viability, migration and tight junction protein expression, underscoring M2's indirect protective role. Importantly, additional IL‐10 neutralization experiments further strengthened the mechanistic interpretation of the co‐culture system. Functional blockade of IL‐10 partially attenuated the protective effects of conditioned medium from NaB‐treated macrophages on injured Caco‐2 cells, including impaired wound healing, reduced epithelial viability and diminished restoration of tight junction‐associated proteins. These findings suggest that macrophage‐derived IL‐10 is an important contributor to the epithelial protective effects induced by NaB.

Collectively, the strengths of the present study include the integration of multiple experimental systems, including in vivo CDI models, primary macrophages, immortalized cell lines and macrophage–epithelial co‐culture models, together with comprehensive evaluation of epithelial barrier function and inflammatory responses. At the same time, several limitations should be acknowledged. First, the current findings are derived primarily from preclinical models and therefore require further validation in human systems. Second, although the data consistently support a potential role for PI3K/Akt/mTOR signalling in NaB‐mediated immunomodulation, definitive mechanistic confirmation will require future studies employing genetic approaches, such as RNA interference, CRISPR‐based gene editing, or conditional knockout strategies targeting pathway‐specific components. Such investigations will be important for clarifying the precise molecular mechanisms underlying NaB‐mediated protection during CDI.

## 5 Conclusion

Low‐dose NaB exerts potent protective effects against CDI by integrating immune regulation with epithelial barrier preservation. NaB promotes macrophage polarization towards an M2 phenotype, enhances IL‐10‐dependent macrophage–epithelial communication and attenuates intestinal inflammation while accelerating mucosal repair. These effects are associated with modulation of PI3K/Akt/mTOR signalling, identifying this pathway as an important component of NaB‐mediated immunometabolic regulation. By linking macrophage reprogramming to epithelial protection, our findings provide mechanistic insight into how microbiota‐derived metabolites shape host responses during CDI and support the development of NaB‐based host‐directed therapeutic strategies for intestinal inflammatory diseases.

## Author Contributions

Sisi Zhang is the first author and conceived the project, designed all experiments, and wrote the manuscript independently, while Jingxiang Zhou assisted in some of the animal and cell experiments. Caixia Tan assisted with the ethical review. Yuanyuan Xiao, Ju Zou, and Biyue Tian provided technical guidance on the experiment. Chunhui Li and Anhua Wu supervised the completion of the manuscript and contributed equally as corresponding authors. All authors read and approved the final manuscript.

## Funding

This work was supported by the National Key Research and Development Program of China (No. 2022YFC2009801; No. 2022YFC2009805), Changsha science and technology plan project (No. kq2202059) and Scientific and technological personnel lifting Project in Hunan Province (No. 2023TJ‐Z11), the Natural Science Foundation of Hunan Province (No. 2025JJ50648), the National Natural Science Foundation of China—China State Railway Group Co. Ltd. Railway Fundamental Research Joint Fund (No. U2368213), Health Economics of Active Screening for Prevention and Control of Multidrug‐Resistant Bacterial Infections (No. GY2023025), National High‐Level Medical Talent Program National Outstanding Young Physician Program.

## Ethics Statement

All animal experiments were conducted in accordance with the ARRIVE guidelines 2.0 and were approved by the Animal Ethics Committee of the Xiangya Hospital, Central South University (Changsha, China; licence code: 202511196). The manuscript does not contain clinical studies or patient data.

## Conflicts of Interest

The authors declare no conflicts of interest.

## Supporting information


**Figure S1:** Dose‐dependent effects of sodium butyrate on cell viability and epithelial barrier‐associated protein expression. (A–D) Cell viability of Caco‐2 cells, THP‐1‐derived macrophages, RAW264.7 macrophages and BMDM, respectively, after 24 h exposure to increasing concentrations of sodium butyrate (NaB). Low‐dose NaB (0.01–1 mM) showed minimal cytotoxicity and modestly enhanced cell viability, whereas higher concentrations (≥ 5 mM) significantly reduced viability in a dose‐dependent manner. Data are presented as mean ± SD. Statistical significance is indicated as *p* < 0.05, *p* < 0.01, *p* < 0.001 and *p* < 0.0001. (E) Representative immunofluorescence staining of Occludin (red) and nuclei (DAPI, blue) in Caco‐2 cells treated with different concentrations of NaB. Low‐dose NaB enhanced junctional Occludin localization, whereas high‐dose NaB markedly attenuated Occludin expression. Scale bar, 20 μm. (F) Representative Western blot analysis of ZO‐1 and Occludin protein expression in Caco‐2 cells following NaB treatment. Consistent with immunofluorescence findings, low‐dose NaB promoted epithelial barrier‐related protein expression, whereas higher concentrations resulted in progressive suppression of ZO‐1 and Occludin levels. β‐actin served as the loading control.
**Figure S2:** PMA (100 ng/mL) induces differentiation of human THP‑1 monocytic cells into adherent macrophage‑like cells in vitro. (A) Representative bright‑field micrographs showing morphological changes of THP‑1 cells with or without 100 ng/mL PMA stimulation. Cellular images were captured sequentially under 10×, 20× and 40× magnification. Untreated THP‑1 monocytes maintained round morphology and suspension growth; in contrast, PMA‑treated cells became adherent with irregular, stretched macrophage‑like cellular shape, indicating monocyte‑to‑macrophage differentiation. (B) Western blot analysis of macrophage differentiation biomarkers CD11b and CD68 in THP‑1 whole‑cell lysates from vehicle control (−) and PMA‑treated (+) groups. β‑actin was probed as endogenous loading control for equal sample normalization. Robust upregulation of CD11b (128 kDa) and CD68 (38 kDa) protein expression was observed after PMA incubation, biochemically validating successful THP‑1 macrophage differentiation.
**Figure S3:** IL‐10 mediates the epithelial reparative effects of conditioned medium from NaB‐treated macrophages. (A) Representative wound‐healing images of Caco‐2 monolayers at 0 and 24 h following treatment with conditioned medium (CM) derived from NaB‐treated THP‐1 macrophages in the presence or absence of IL‐10 neutralization, recombinant human IL‐10 (rhIL‐10), IL‐10Rα blockade, or corresponding isotype controls. (B) Representative immunofluorescence staining of Occludin (red) and nuclei (DAPI, blue) showing epithelial barrier integrity under the indicated conditions. Neutralization of IL‐10 or blockade of IL‐10Rα impaired CM‐mediated wound closure and Occludin restoration, whereas rhIL‐10 supplementation rescued these effects.

## Data Availability

Data sharing not applicable to this article as no datasets were generated or analysed during the current study.
