# Supplementary material for: Immunometabolic Modulation by Low‐Dose Sodium Butyrate: PI3K/Akt/mTOR Signalling Orchestrates Macrophage Polarization to Protect Against Clostridioides difficile Colitis
Source: Immunology. 2026 Aug 11;179(4):594–608. doi: 10.1111/imm.70179 (PMC13725437; doi:10.1111/imm.70179)
Supplement: Supplementary file 1 [file IMM-179-594-s001.docx]

**Supplementary Figures**

Supplementary Figure 1. Dose-dependent effects of sodium butyrate on cell viability and epithelial barrier-associated protein expression.

(A–D) Cell viability of Caco-2 cells, THP-1-derived macrophages, RAW264.7 macrophages and BMDM, respectively, after 24 h exposure to increasing concentrations of sodium butyrate (NaB). Low-dose NaB (0.01–1 mM) showed minimal cytotoxicity and modestly enhanced cell viability, whereas higher concentrations (≥5 mM) significantly reduced viability in a dose-dependent manner. Data are presented as mean ± SD. Statistical significance is indicated as P < 0.05, P < 0.01, P < 0.001, and P < 0.0001. (E) Representative immunofluorescence staining of Occludin (red) and nuclei (DAPI, blue) in Caco-2 cells treated with different concentrations of NaB. Low-dose NaB enhanced junctional Occludin localization, whereas high-dose NaB markedly attenuated Occludin expression. Scale bar, 20 μm. (F) Representative Western blot analysis of ZO-1 and Occludin protein expression in Caco-2 cells following NaB treatment. Consistent with immunofluorescence findings, low-dose NaB promoted epithelial barrier-related protein expression, whereas higher concentrations resulted in progressive suppression of ZO-1 and Occludin levels. β-actin served as the loading control.

Supplement Figure 2. PMA (100ng/ml) induces differentiation of human THP‑1 monocytic cells into adherent macrophage‑like cells in vitro.

(A) Representative bright‑field micrographs showing morphological changes of THP‑1 cells with or without 100 ng/mL PMA stimulation. Cellular images were captured sequentially under 10×, 20× and 40× magnification. Untreated THP‑1 monocytes maintained round morphology and suspension growth; in contrast, PMA‑treated cells became adherent with irregular, stretched macrophage‑like cellular shape, indicating monocyte‑to‑macrophage differentiation. (B) Western blot analysis of macrophage differentiation biomarkers CD11b and CD68 in THP‑1 whole‑cell lysates from vehicle control (−) and PMA‑treated (+) groups. β‑actin was probed as endogenous loading control for equal sample normalization. Robust upregulation of CD11b (128 kDa) and CD68 (38 kDa) protein expression was observed after PMA incubation, biochemically validating successful THP‑1 macrophage differentiation.

Supplementary Figure 3. IL-10 mediates the epithelial reparative effects of conditioned medium from NaB-treated macrophages.

(A) Representative wound-healing images of Caco-2 monolayers at 0 and 24 h following treatment with conditioned medium (CM) derived from NaB-treated THP-1 macrophages in the presence or absence of IL-10 neutralization, recombinant human IL-10 (rhIL-10), IL-10Rα blockade, or corresponding isotype controls. (B) Representative immunofluorescence staining of Occludin (red) and nuclei (DAPI, blue) showing epithelial barrier integrity under the indicated conditions. Neutralization of IL-10 or blockade of IL-10Rα impaired CM-mediated wound closure and Occludin restoration, whereas rhIL-10 supplementation rescued these effects.
